# Effect of nutritive and non-nutritive sweeteners on hemodynamic responses to acute stress: a randomized crossover trial in healthy women

**DOI:** 10.1038/s41387-019-0104-y

**Published:** 2020-01-02

**Authors:** Jérémy Cros, Lucie Bidlingmeyer, Robin Rosset, Kevin Seyssel, Camille Crézé, Nathalie Stefanoni, Philippe Schneiter, Luc Tappy

**Affiliations:** Faculty of Biology and Medicine, Department of Biomedical Sciences, University of Lausanne, 1015 Lausanne, Switzerland

**Keywords:** Obesity, Cardiovascular diseases, Cardiovascular diseases

## Abstract

**Background:**

The mechanisms by which chronic stress increases the risk of non-communicable diseases remain poorly understood. On one hand, chronic stress may increase systemic vascular resistance (SVR) and blood pressure, which may lead to blood vessels injury and altered myocardial perfusion. On the other hand, chronic stress may promote the overconsumption of sugar-containing foods and favor obesity. There is indeed evidence that sweet foods are preferentially consumed to alleviate stress responses. The effects of nutritive and non-nutritive sweeteners (NNS) on hemodynamic stress responses remain however largely unknown.

**Objective/design:**

This study aimed at comparing the effects of sucrose-containing and NNS-containing drinks, as compared to unsweetened water, on hemodynamic responses to acute stress in twelve healthy female subjects. Acute stress responses were elicited by a 30-min mental stress (5-min Stroop’s test alternated with 5-min mental arithmetic) and a 3-min cold pressure test (CPT), each preceded by a resting baseline period. Hemodynamic stress responses were investigated by the repeated measurement of mean arterial pressure and the continuous monitoring of cardiac output by thoracic electrical bioimpedance measurement. SVR was selected as a primary outcome because it is a sensitive measure of hemodynamic responses to acute stress procedures.

**Results:**

With all three drinks, SVR were not changed with mental stress (*P* = 0.437), but were increased with CPT (*P* = 0.045). Both mental stress and CPT increased mean arterial pressure and heart rate (all *P* < 0.001). Cardiac output increased with mental stress (*P* < 0.001) and remained unchanged with CPT (*P* = 0.252). No significant differences in hemodynamic responses were observed between water, sucrose and NNS (stress × condition, all *P* *>* 0.05).

**Conclusions:**

These results demonstrate that sucrose and NNS do not alter hemodynamic responses to two different standardized acute stress protocols.

## Introduction

Psychosocial stress is currently suspected to play an important role in the development of non-communicable diseases^1^. This is notably based on observations that both acute and chronic stress can favor consumption of energy-dense foods^2^, and that stress-induced glucocorticoid secretion may promote visceral fat deposition^3^, thus leading in the long term to the development of abdominal obesity, insulin resistance and increased cardiometabolic risk^4^. It has also been proposed that hemodynamic responses to acute stress may cause acute myocardial ischemia^5,6^. Interestingly, sucrose consumption has been reported to reduce sympatho-adrenal activation and hemodynamic responses to acute stress in rodents^7^ and humans^8,9^. It has further been postulated that exposure to chronic stress may trigger the consumption of sweet foods in order to alleviate stress responses^10^. Such stress-induced eating behavior may however be associated with a risk of energy overconsumption and obesity^11^.

In rats, sucrose-nutritive and non-nutritive sweeteners (NNS) consumption has been associated with a down-regulation of corticotropin-releasing factor (CRF) expression in the brain^12^. It was therefore proposed that the non-metabolic properties of sweet foods were instrumental in this effect, likely by stimulating the release of intracerebral opioid^13,14^. However, one study reported that sucrose, but not saccharin, decreased intracerebral CRF secretion in adrenalectomized rats, suggesting that energy-dependent metabolic effects of sucrose were also involved^15^. This hypothesis was further supported by a human study showing that salivary cortisol response to mental stress was decreased after a high-sucrose diet, but increased after a NNS-containing diet^16^.

The effects of nutritive and non-nutritive sweeteners on hemodynamic responses to acute stress remain to date highly unknown. In order to address this issue, we submitted a group of healthy women to a mental stress, which elicits predominant β-adrenergic hemodynamic responses^17^, and to a cold pressure test (CPT), which elicits predominant α-adrenergic responses^18^, together with repeated oral administration of sucrose-sweetened or NNS-sweetened drinks or of unsweetened water.

## Materials and methods

### Ethics

The experimental part of this study was conducted between September 2015 and October 2016. This study was performed in accordance with the 1983 revision of the Declaration of Helsinki and was approved by the Human Research Ethics Committee of Canton de Vaud. All participants were fully informed of the nature and risks involved by the procedures and provided informed, written consent. This study was registered at clinicaltrial.gov as NCT02973334.

### Participants

**Fig. 1:**
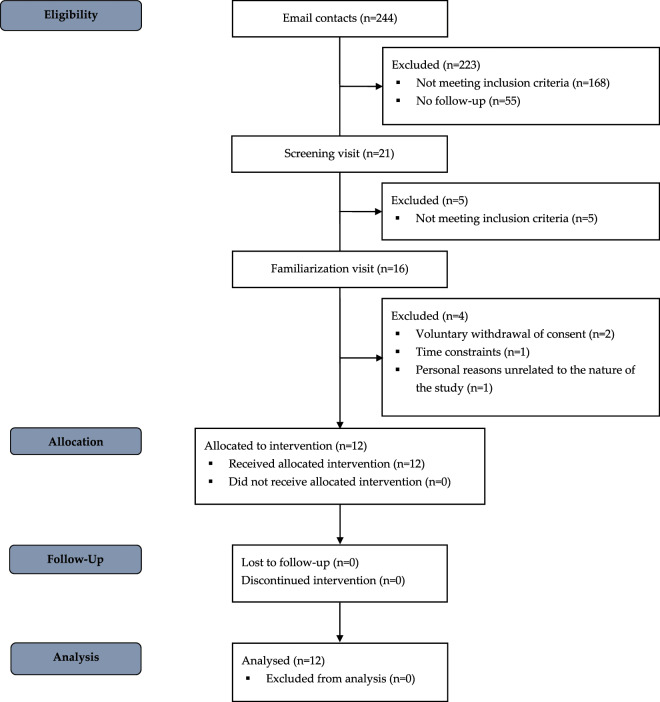
Participants' flow diagram. Eligibility, allocation, follow-up and analysis of study participants.

Eligibility, allocation, follow-up and analysis of participants are depicted in Fig. 1. This study included sixteen participants who were recruited by advertisement at local university campuses. All were Caucasian females, 18 to 40 years old, with a body mass index between 18.5 and 25 kg × m^−2^ and used monophasic oral contraceptive agents. Exclusion criteria were blood pressure >140/90 mmHg, history or current psychological and cardiovascular disorders, anemia or history of anemia, daily consumption of >5 dL caloric and NNS sweetened-beverages, >400 mg caffeine, >10 g alcohol, color blindness, body weight variation >3 kg over the past 4 weeks, performing physical activity >4 h.week^−1^, any current medical treatment, current smokers, or consumption of illicit drugs. Eligible participants were invited to a familiarization visit to be exposed to the mental stress and CPT procedures, as conducted during the functional evaluations. Twelve female participants were finally enrolled and completed the study (mean ± SD age: 21.8 ± 2.4 years; body mass index: 21.6 ± 1.7 kg × m^−2^).

### Study design

Each participant took part to three functional evaluations, during which they consumed either water-containing, or sucrose-containing or NNS-containing test drinks, according to a randomized, crossover design. Successive visits were separated by 1–3-week washout periods. Participants received instructions to consume an energy-balanced, weight-maintenance diet during the two days preceding each functional evaluation. They were asked to avoid sugars-rich and NNS-rich foods, and to abstain from consuming caffeine and alcohol during this pre-evaluation period. Participants were also instructed to have a minimal 8 h sleep opportunity time per night and to refrain from structured physical activity. Compliance was checked using food, physical activity, and sleep records.

For each functional test, participants reported at 0700 to the Metabolism, Nutrition, and Physical Activity unit from the Clinical Research Center of the Lausanne University Hospital. They had been fasting since 2200 the day before. Upon arrival, they were asked to void and were weighed (Seca 708, Seca GmbH, Hamburg, Germany). They were then comfortably installed in a bed in a reclining position and four dual hemodynamic sensors (BioZ AdvaSense, Vermed, Buffalo, New York, United States) were placed to enable thoracic electrical bioimpedance measurements. Briefly, two sensors were positioned on each side of the neck while two others were placed on midaxillary lines at the level of the xiphoid process.

**Fig. 2:**
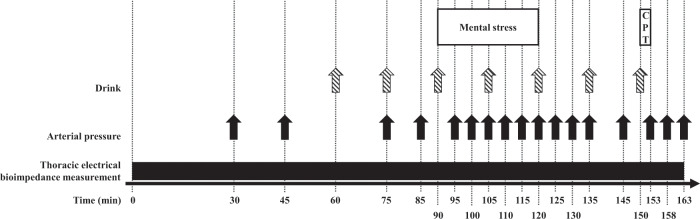
Illustration of the functional evaluation. After a 2-day prescribed, weight-maintenance diet, 12 participants were studied on 3 occasions, according to a randomized, crossover design. Briefly, after an overnight fast, the functional evaluation started with a 90-min resting period. It was followed by a 30-min mental stress (consisting of 5-min periods of Stroop’s color word conflict test alternated with 5-min periods of complex mental arithmetic operations), a 30-min recovery period and a 3-min CPT. From time 60, and every 15 min until the end of the test, participants ingested 25-mL test drinks containing either pure water, 106 g.L^−1^ sucrose or a mix of NNS (392 mg.L^−1^ sodium cyclamate, 181 mg.L^−1^ acesulfame K and 116 mg.L^−1^ aspartame) after a 10-s mouth rinse. The effects of test drinks on hemodynamic stress responses were investigated by the repeated measurement of mean arterial pressure, as well as the continuous monitoring of cardiac output by thoracic electrical bioimpedance measurement. Each functional evaluation was separated by 1–3-week washout periods. CPT: cold pressure test.

At 0800 (time 0 min), the functional evaluation started with a 90-min resting period (Fig. 2). It was followed by a 30-min mental stress (time 90–120 min), consisting of 5-min periods of Stroop’s color word conflict test alternated with 5-min periods of complex mental arithmetic operations^19^. Thereafter, a 30-min recovery period was allowed, and a 3-min CPT was carried out at time 150 min. CPT consisted in immersing their right hand for 3 min in ice water^18^. From time 60 min, and every 15 min until the end of the test (time 60, 75, 90, 105, 120, 135, and 150 min), participants ingested 25-mL test drinks containing either water (Water condition), 106 g.L^−1^ sucrose (Hänseler AG, Herisau, Switzerland; Sucrose condition) or a mix of 392 mg.L^−1^ sodium cyclamate (INRESA, Bartenheim, France), 181 mg.L^−1^ acesulfame K (Celanese Corporation, Sulzbach, Germany), and 116 mg.L^−1^ aspartame (INRESA, Bartenheim, France) (NNS condition), based on the composition of commercial drinks^20^. Before swallowing every 25-mL test drink, participants were instructed to rinse their mouth for 10 s with it to activate oral sweet taste receptors.

Mean arterial pressure (calculated as 1/3 systolic blood pressure + 2/3 diastolic blood pressure) was recorded at time 30, 45, 75, 85, 95, 100, 105, 110, 115, 120, 125, 130, 135, 145, 153, 158, and 163 min using an automatic sphygomomanometer (BioZ ICG Monitor, CardioDynamics, San Diego, California, United States), positioned on the volunteers’ left arm. In order to obtain accurate values, arterial pressure was measured in triplicate at each time points and the averages were retained. Throughout the study, cardiac output was measured by thoracic electrical bioimpedance using a BioZ impedance cardiograph (model BZ-4110-221; CardioDynamics, San Diego, California, United States). SVR was calculated as proposed by the manufacturer, i.e.,:

$$\begin{array}{lcc}{\mathrm{SVR}}\,=\\\,\,\,\,\,\,\,\,\,\,\,\,\,\,\,\,\,\,\,\,\,\,80\,\times \frac{{\left({{\mathrm{mean}}\,{\mathrm{arterial}}\,{\mathrm{pressure-central}}\,{\mathrm{venous}}\,{\mathrm{pressure}}} \right)}}{{{\mathrm{cardiac}}\,{\mathrm{output}}}}\end{array}$$

where the default value for central venous pressure was 6 mmHg.

### Data handling and statistical analyses

The primary outcome of this study was SVR changes across drink conditions (i.e., Water, Sucrose, and NNS). A sample size of *n* = 12 participants was calculated (1-β: 90%; *α* = 0.05) to detect a 6 ± 4% difference in postprandial SVR after the ingestion of sucrose, compared with water, as previously reported^21^. The sequence of treatment allocation was determined by random and balanced generation of six-sequence blocks using Microsoft Excel software (Microsoft Corp., Redmond, WA, USA). Hemodynamic data were averaged by periods, i.e., before drink ingestion (time 0–60 min), “baseline pre-mental stress” (time 60–85 min), “mental stress” (time 90–120 min), “baseline pre-CPT” (time 125–145 min), and “CPT” (time 150–153 min). Prior to statistical analysis, data distribution and equality of variances were checked using Shapiro-Wilk and Bartlett tests, respectively. Non-normally distributed data (i.e., SVR before drink ingestion and during the baseline pre-CPT period) were transformed using the Box-Cox algorithm_._ Effects of acute stress, condition and their interaction were investigated using mixed-model analyses. *P* values *<* 0.05 were considered as significant. Statistical analyses were performed using the R software, version 3.3.1 (R foundation for Statistical Computing, Vienna, Austria). Data are presented as mean ± SD.

## Results

### Body weight and hemodynamic parameters before drink ingestion (time 0–60 min)

Participants showed no significant differences in body weight between the 3 experimental conditions (Table 1). SVR, mean arterial pressure, cardiac output and heart rate were all similar before drink ingestion (Table 1).

**Table 1:** Body weight and hemodynamic parameters before drink ingestion.

	Water	Sucrose	NNS	*P*
Body weight (kg)	59.2 ± 4.7	59.1 ± 4.5	59.3 ± 4.6	0.678
Resting systemic vascular resistance (U)	1140 ± 213	1135 ± 192	1208 ± 254	0.505
Resting mean arterial pressure (mmHg)	66 ± 6	65 ± 6	66 ± 6	0.322
Resting cardiac output (L × min^−1^)	5.0 ± 1.0	4.9 ± 0.8	4.7 ± 0.8	0.416
Resting heart rate (beats × min^−1^)	69 ± 10	66 ± 10	67 ± 9	0.223

Values are expressed as mean ± SD. The normality and homoscedatiscity of data distribution were insepected by Shapiro-Wilk and Bartlett tests, respectively. For statistical analyses, the Box-Cox algorithm was applied to systemic vascular resistance. Differences between conditions were investigated using mixed-model analyses, with condition as fixed factor and participant-specific intercepts and slopes as random effects. All data are based on *n* = 12 participants

*P* < 0.05 was considered as statistically significant

*NNS* non-nutritive sweetener, *U* arbitrary unit

### Effects of a 30-min mental stress

**Fig. 3:**
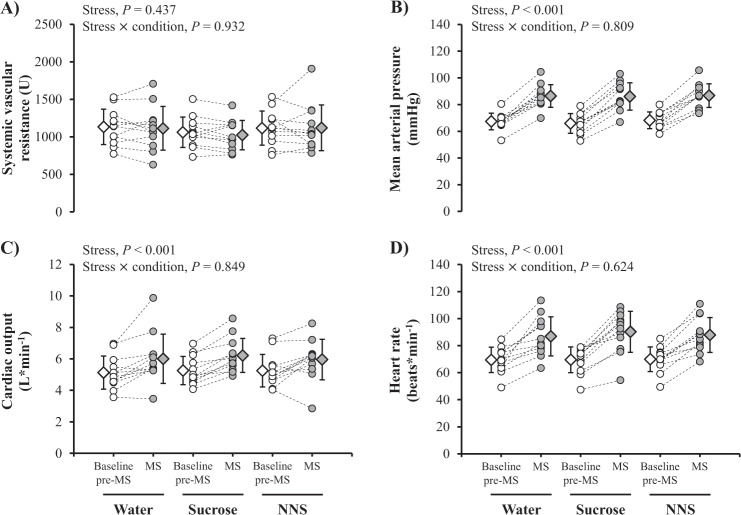
Individual (dots with dashed lines) and mean (±SD; diamonds) changes in systemic vascular resistance (**a**), mean arterial pressure (**b**), cardiac output (**c**), and heart rate (**d**) after a 30-min mental stress with the consumption of water-containing, sucrose-containing, or NNS-containing test drinks. The normality and homoscedatiscity of data distribution were insepected by Shapiro-Wilk and Bartlett tests, respectively. Effects of mental stress and its interaction with drink condition were investigated using mixed-model analyses, with mental stress and drink condition as fixed factors and participant-specific intercepts and slopes as random effects. All data are based on *n* = 12 participants. *P* < 0.05 was considered as statistically significant. Baseline pre-MS, baseline pre-mental stress (time 60–85 min; white symbols); MS mental stress (time 90–120 min; gray symbols), NNS non-nutritive sweetener, U arbitrary unit.

Hemodynamic parameters during the baseline pre-mental stress period (time 60–85 min) were all similar between conditions (SVR, *P* = 0.422; mean arterial pressure, *P* = 0.185; cardiac output, *P* = 0.805; heart rate, *P* = 0.932; Fig. 3). Mental stress did not change SVR (Fig. 3a), but significantly increased mean arterial pressure (Fig. 3b), cardiac output (Fig. 3c) and heart rate (Fig. 3d). Changes in hemodynamic parameters during mental stress were not altered by drink conditions (all stress × condition interactions, *P* *>* 0.05).

### Effects of a 3-min CPT

**Fig. 4:**
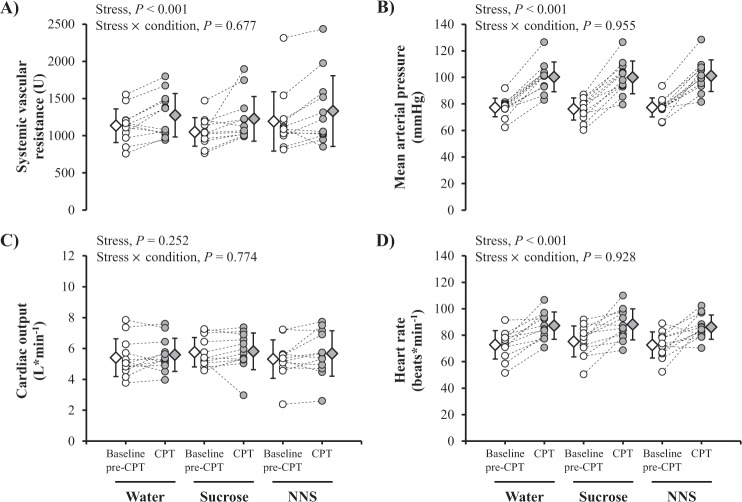
Individual (dots with dashed lines) and mean (±SD; diamonds) changes in systemic vascular resistance (**a**), mean arterial pressure (**b**), cardiac output (**c**), and heart rate (**d**) after a 3-min CPT with the consumption of water-containing, sucrose-containing, or NNS-containing test drinks. The normality and homoscedatiscity of data distribution were insepected by Shapiro-Wilk and Bartlett tests, respectively. For statistical analyses, the Box-Cox algorithm was applied to systemic vascular resistance. Effects of CPT and its interaction with drink condition were investigated using mixed-model analyses, with CPT and drink condition as fixed factors and participant-specific intercepts and slopes as random effects. All data are based on *n* = 12 participants. *P* < 0.05 was considered as statistically significant. Baseline pre-CPT, baseline pre-cold pressure test (time 125–145 min; white symbols); CPT cold pressure test (time 150–153 min; gray symbols); NNS non-nutritive sweetener, U arbitrary unit.

Hemodynamic parameters during the baseline pre-CPT period (time 125–145 min) were all similar between conditions (SVR, *P* = 0.202; mean arterial pressure, *P* = 0.448; cardiac output, *P* = 0.235; heart rate, *P* = 0.230; Fig. 4). CPT significantly increased SVR (Fig. 4a), mean arterial pressure (Fig. 4b) and heart rate (Fig. 4d), but did not change cardiac output (Fig. 4c). Changes in hemodynamic parameters during CPT were not altered by drink conditions (all stress × condition interactions, *P* *>* 0.05).

## Discussion

The aim of this study was to investigate whether sweet drinks change hemodynamic stress responses. To test this hypothesis, twelve healthy women were sequentially exposed to a 30-min mental stress and a 3-min CPT, i.e., two stress procedures which both stimulate SNS activity, but elicit different hemodynamic responses through differential activations of β-adrenergic or α-adrenergic receptors. Hemodynamic responses were monitored on three separate occasions, with subjects consuming either water-containing, or sucrose-containing or NNS-containing drinks. We selected SVR as a primary outcome (measured by thoracic electrical bioimpedance measurement), because this parameter is sensitively altered by acute stress procedures^17,18^. Mental stress and CPT both produced the expected effects on hemodynamic responses. No significant differences were observed between the three experimental conditions, however, suggesting that neither sucrose and nor NNS alter stress-induced stimulation of SNS.

### Validity of acute stress procedures

Mental stress elicited hemodynamic responses similar to those reported by other studies published in the literature, including increases of heart rate, mean arterial pressure, and cardiac output, and either no change^22,23^ or a slight decrease^17,24–26^ of SVR. Experimental studies have further demonstrated that the effects of mental stress on cardiac output and SVR were in part mediated by a β-adrenergic, nitric oxide dependent vasodilation in skeletal muscle^27^.

CPT also elicited hemodynamic responses similar to those reported in the literature, i.e., significant increases of SVR and heart rate, and mean arterial pressure, without any change in cardiac output^18,28^.

### Effect of sucrose and NNS on hemodynamic responses

As compared to Water, Sucrose, and NNS did not change any of the hemodynamic parameters which were monitored during acute mental stress and CPT. This is indirect evidence that sweeteners, whether containing calories or not, did not alter the sympathetic activation of heart rate during acute mental stress and CPT, did not enhance cardiac muscle contraction strength, and did not induce peripheral (muscle) vasodilation during acute mental stress or peripheral vasoconstriction during CPT. This observation appears at odds with several other studies, however. In human newborns, sucrose ingestion before painful procedures (e.g., heel prick), has indeed been associated with reduced heart rate^8,9^, as well as alleviation of several pain indicators^29,30^. These effects were reported when sucrose was intraorally delivered, but not after intragastric administration, suggesting that they were linked to taste-related mechanisms^31^. This observation was further supported by other pediatric studies showing that pain indicators were effectively reduced with solutions containing sucrose and aspartame, but not polycose (a glucose polymer that is metabolized by humans, but that has a very low sweetness)^32^. The reasons for these discrepancies between children and adults remain unknown, but may be due to developmental changes in sweet taste sensitivity^33^ or in actions of sweet taste receptors on cerebral neuronal circuits^34^, leading to different effects on hemodynamic stress responses.

### Practical implications

There is currently much concern that stress may be involved in the development of non-communicable diseases^1^, but the responsible mechanisms remain debated. Among other hypotheses, it has been proposed that chronic stress may drive an overconsumption of sugar-rich foods^2^ as a mean to alleviate stress-related neuroendocrine and hemodynamic responses^7–10,12,15,16^. The present observations demonstrate that limited and repeated consumption of sucrose or NNS are both ineffective in modulating hemodynamic responses to two different standardized acute stress procedures. However, this study does by no means disprove the hypothesis that acute stress may promote the consumption of sweet or energy-dense foods through other unrelated mechanisms. This also do not challenge the concept that repeated acute stress exposure may contribute to the pathogenesis of non-communicable diseases by increasing mean blood pressure, altering myocardial perfusion^5,6^, or increasing cortisol secretion^4^, which may in the long term lead to vascular damages and metabolic dysfunction.

### Limitations

This study has some limitations that need to be addressed. First, we investigated the effects of acute stress procedures on hemodynamic parameters, but we did not directly assess SNS activity by muscle sympathetic nerve activity or norepinephrine-spillover measurements, nor the activity of the hypothalamic-pituitary-adrenal axis. Second, we aimed at designing this study with the highest ecological validity, and therefore asked participants to swallow the test drinks after their mouth rinses. As a consequence, we cannot discard that small changes in insulin concentrations induced by glucose metabolism may have altered hemodynamic responses through its vasodilatory properties^21,35^. We can also not rule out that the hemodynamic responses to CPT may have been mitigated by an unintentional performance of a Valsalva maneuver^36^. Finally, this study was conducted on young, healthy women, and these results cannot be extrapolated to obese individuals or to men, who showed substantial differences in hemodynamic stress responses^19,37^.

## Conclusion

In summary, these results demonstrate that consumption of drinks sweetened with sucrose or NNS do not alter hemodynamic responses to standardized acute stress protocols.
